# Racial and Ethnic Disparities in Insurance Coverage Among US Adults Aged 60 to 64 Years

**DOI:** 10.1001/jamanetworkopen.2022.9406

**Published:** 2022-04-28

**Authors:** Anna Patterson, Taylor J. Robinson, Eric T. Roberts

**Affiliations:** 1 Department of Health Policy and Management, University of Pittsburgh Graduate School of Public Health, Pittsburgh, Pennsylvania; 2 Department of Epidemiology University of Pittsburgh Graduate School of Public Health, Pittsburgh, Pennsylvania

## Abstract

This cross-sectional study examines racial and ethnic disparities in health insurance coverage among Black and Hispanic adults vs White adults aged 60 to 64 years.

## Introduction

Congress is weighing several health policy reforms, motivated partly by the goal of reducing racial and ethnic disparities in insurance coverage.^1,2^ Since implementation of the Patient Protection and Affordable Care Act (ACA), coverage disparities have narrowed, yet non-Hispanic Black and Hispanic adults remain more likely to be uninsured than non-Hispanic White adults (hereafter Black, Hispanic, or White), particularly in states that did not expand Medicaid under the ACA.^3^ Adults in nonexpansion states are ineligible for ACA Marketplace subsidies if their incomes are less than 100% of the federal poverty level (FPL).^3^

One reform under consideration by Congress, termed Medicare-for-more, would lower the age threshold for Medicare to age 60 years from 65 years.^2^ If enacted, this policy would extend near-universal insurance to a larger share of the US population. We assessed potential for expanding Medicare to reduce existing coverage disparities among Black and Hispanic adults vs White adults aged 60 to 64 years. We hypothesized that sizeable coverage disparities remain, particularly among adults with low income in Medicaid nonexpansion states, which could be reduced through an expansion of Medicare.

## Methods

We analyzed the 2019 American Community Survey, which reports demographic, residential, and insurance information. We used self-reported race and ethnicity to categorize respondents as Black, Hispanic, or White. The University of Pittsburgh institutional review board waived study review and informed consent because this analysis used deidentified data. We followed Strengthening the Reporting of Observational Studies in Epidemiology (STROBE) reporting guideline.

We conducted 2 analyses of uninsurance and racial and ethnic coverage disparities among adults aged 60 to 64 years, illustrating potential for near-universal Medicare coverage to reduce these disparities. First, we estimated national and state-level uninsured rates among Black and Hispanic adults of all incomes. We estimated state-level rates by fitting linear regression models estimating uninsurance as a function of state fixed effects. Second, among adults with incomes less than 138% FPL, we compared Black-White and Hispanic-White disparities in uninsurance between Medicaid expansion and nonexpansion states by fitting linear models to estimate uninsurance as a function of expansion status, race and ethnicity, and the interaction of these variables. All models adjusted for sex, marital status, employment status income, disability, and used ACS survey weights (eAppendix in the Supplement). Expansion status was assessed as of December 31, 2019.

## Results

Among US adults aged 60 to 64 years, 52.1% (95% CI, 52.0%-52.4%) were female, 12.3% (95% CI, 12.1%-12.5%) were Black, 11.6% (95% CI, 11.4%-11.8%) were Hispanic, and 76.1% (95% CI, 75.9%-76.4%) were White. Among adults aged 60 to 64 years who were uninsured in 2019 across all states and income levels, 6.9% (95% CI, 5.8%-8.0%) were Black, 15.1% (95% CI, 11.1%-19.2%) were Hispanic, and 7.0% (95% CI, 6.0%-8.1%) were White. Uninsurance among Black adults exceeded 10% in 17 states, and among Hispanic adults it exceeded 10% in 38 states (Figure).

**Figure.**
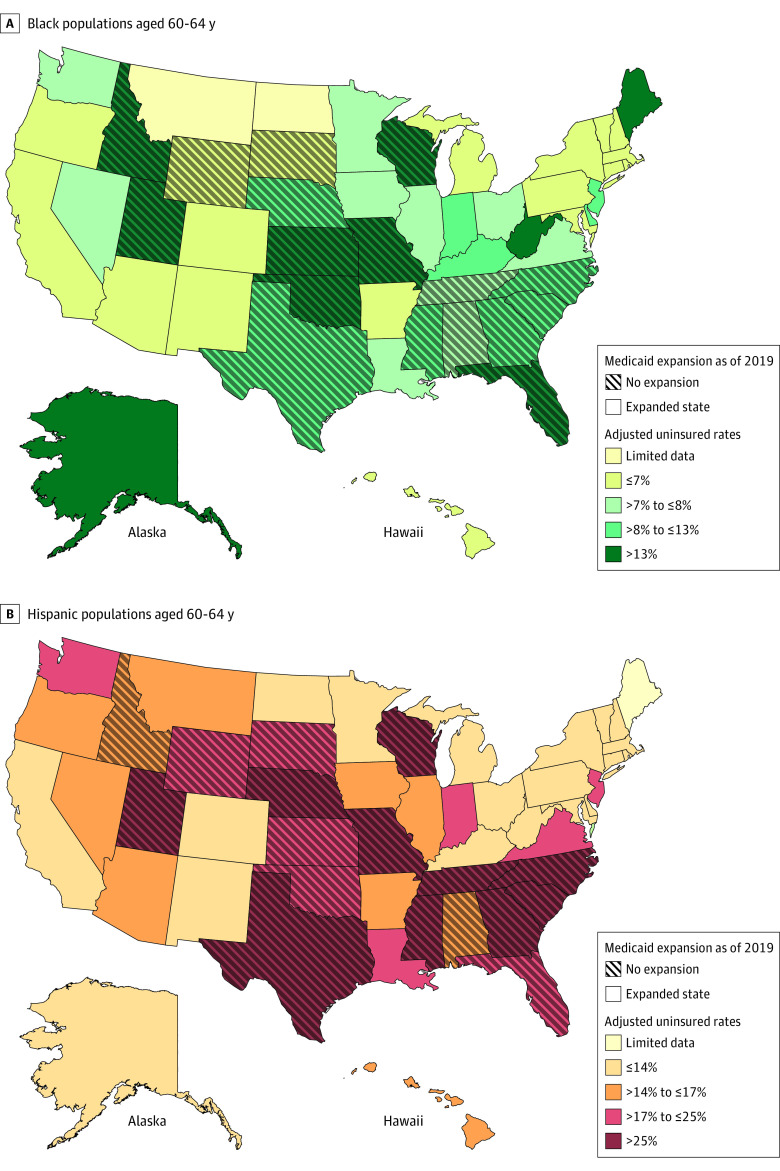
State-Level Uninsured Rates Among Black and Hispanic Adults Aged 60 to 64 Years Across All Income Levels Figures show adjusted uninsurance rates among non-Hispanic Black adults (A) and Hispanic adults (B) aged 60 to 64 years by state, pooled across all income levels. Estimates adjusted for sex, marital status, income, disability status, employment status and weighted by survey weights provided with the American Community Survey. Uninsured rates for a given racial or ethnic group are not shown for states with observations fewer than 207 for that group (among adults aged 60-64 years) and indicated on the map as limited data. Maps were generated using ArcGIS Pro. States that did not expand Medicaid by December 31, 2019, have a crosshatched pattern (18 nonexpansion states). As of October 2021, 12 states had not expanded Medicaid: North Carolina, South Carolina, Florida, Georgia, Alabama, Tennessee, Mississippi, Texas, Kansas, Wyoming, South Dakota, and Wisconsin.

In Medicaid expansion states, 18.0% (95% CI, 14.5%-21.6%) of Hispanic adults and 11.5% (95% CI, 9.7%-13.3%) of White adults aged 60 to 64 years with incomes less than 138% FPL were uninsured, representing a coverage disparity of 6.6 percentage points (95% CI, 2.7-10.5 percentage points; *P* < .001) (Table). In nonexpansion states, 42.6% (95% CI, 35.7%-49.6%) of Hispanic adults and 24.7% (21.7%-27.7%) of White adults aged 60 to 64 years with incomes less than 138% FPL were uninsured. Thus, the coverage disparity between low-income Hispanic adults and low-income White adults was 11.4 percentage points larger in nonexpansion states vs expansion states (95% CI, 3.2-19.5 percentage points; *P* = .007). We did not detect significant Black-White coverage disparities in expansion or nonexpansion states.

**Table.** Racial and Ethnic Disparities in Uninsured Rates Among Adults Aged 60 to 64 Years

	Adults, % (95% CI)		
	Black, non-Hispanic	Hispanic	White, non-Hispanic
**Share of uninsured adults and disparities in the uninsured rate by race and ethnicity^a^**			
All United States^b^			
All income levels	6.9 (5.8 to 8.0)	15.1 (11.1 to 19.2)	7.0 (6.0 to 8.1)
Disparity in population uninsured^c^	−0.1 (−1.0 to 0.77)	8.1 (4.8 to 11.4)	[Reference]
Income <138% FPL^d^	16.3 (12.7 to 19.9)	27.4 (18.2 to 36.6)	16.2 (13.2 to 19.2)
Disparity in population uninsured^a^	0.1 (−1.9 to 2.2)	11.2 (4.1 to 18.3)	[Reference]
**Stratifying states by Medicaid expansion status, incomes <138% FPL** ^**d**^ **^,^ ^e^**			
Medicaid expansion states			
% Uninsured	9.6 (7.7 to 11.6)	18.0 (14.5 to 21.6)	11.5 (9.7 to 13.3)
Disparity in population uninsured^c^	−1.8 (−3.9 to 0.22)	6.6 (2.7 to 10.5)	[Reference]
Medicaid non-expansion states			
% Uninsured	23.8 (20.6 to 27.1)	42.6 (35.7 to 49.6)	24.7 (21.7 to 27.7)
Disparity in population uninsured	−0.9 (−5.1 to 3.3)	17.9 (10.9 to 24.9)	[Reference]
Difference in disparity between nonexpansion and expansion states^f^	0.9 (3.3 to 5.1)	11.4 (3.2 to 19.5)	[Reference]

Abbreviations: ACS, American Community Survey; FPL, federal poverty level.

^a^ All estimates adjusted for sex, marital status, income, disability status, employment status and weighted by the survey weights provided with the ACS. The 95% CIs were constructed using robust standard errors clustered by state.

^b^ Estimates based on 228 270 observations in the 2019 American Community Survey. When weighted, this sample represents 19 560 429 individuals in the US population, restricted to non-Hispanic Black, Hispanic, non-Hispanic White adults aged 60 to 64 years.

^c^ The disparity represents the difference in uninsured rates between (1) Black vs White or (2) Hispanic vs non-Hispanic White adults, where White adults were analyzed as the reference group. Estimated from a linear regression model that estimated uninsurance as a function of race and ethnicity, adjusting for covariates and survey weights as described previously.

^d^ Estimates based on 35 535 observations in the 2019 American Community Survey, limited to Black, Hispanic, and White adults aged 60 to 64 years with incomes less than 138% of the federal poverty level. When weighted, this sample represents 3 169 774 individuals in the US population.

^e^ States’ Medicaid expansion status as of December 31, 2019, as reported by the Kaiser Family Foundation.

^f^ Estimated from a linear regression model that estimated uninsurance as a function of state Medicaid expansion status, race and ethnicity, and interactions between race and ethnicity and expansion status. Non-Hispanic White adults were the reference group. The coefficients on the interaction terms give the difference in coverage disparities between either non-Hispanic Black and non-Hispanic White or Hispanic and non-Hispanic White adults in states that did vs did not expand Medicaid by 2019. Estimates were adjusted for covariates and survey weights as described previously.

## Discussion

Our results suggest potential for lowering the threshold for Medicare to 60 years of age to reduce existing coverage disparities among adults aged 60 to 64 years, particularly among low-income Hispanic adults in nonexpansion states. These findings are consistent with a recent study that found reductions in coverage disparities among adults acquiring coverage at Medicare’s current age threshold.^4^ Evidence from the ACA suggests that broader coverage in middle age may yield health gains, including reductions in mortality among low-income adults.^5^

One limitation of our analysis is that it uses 2019 data, the most current, which predates the COVID-19 pandemic. Emerging evidence suggests coverage disparities during the pandemic widened in nonexpansion states.^6^ Thus, our results may conservatively reflect the current extent of coverage disparities among older adults.

## Notes

Supplement. **eAppendix.** Statistical Analyses
