# Supplementary material for: Racial and Ethnic Disparities in Insurance Coverage Among US Adults Aged 60 to 64 Years
Source: JAMA Netw Open. 2022 Apr 28;5(4):e229406. doi: 10.1001/jamanetworkopen.2022.9406 (PMC9051983; doi:10.1001/jamanetworkopen.2022.9406)
Supplement: Supplement. — eAppendix. Statistical Analyses [file jamanetwopen-e229406-s001.pdf]

Supplemental Online Content

Patterson A, Robinson TJ, Roberts ET. Racial and ethnic disparities in insurance coverage among US adults aged 60 to 64 years. *JAMA Netw Open.* 2022;5(4):e229406. doi:10.1001/jamanetworkopen.2022.9406

eAppendix. Statistical Analyses

This supplemental material has been provided by the authors to give readers additional information about their work.

© 2022 Patterson A et al. *JAMA Network Open.*

eAppendix. Statistical Analyses

We conducted two analyses using the 2019 American Community Survey to examine the rate of uninsurance among non-Hispanic Black and Hispanic adults ages 60-64 and assess disparities in uninsurance among non-Hispanic Black, Hispanic, and non-Hispanic White adults.

First, we estimated linear regression models with state fixed effects to estimate the state-level proportions of non-Hispanic Black and Hispanic adults who were uninsured in 2019 (pooled across all income levels). These models had the form:

$$Uninsured_{is}=\beta_0+\beta_1 marst_i+\beta_2 disability_i+\beta_3 employed_i+\beta_4 sex_i+\beta_5 fpl_i + \Sigma_s\beta_{6,s}{*}I(state_{is}=s)+\varepsilon_{is} \qquad (1)$$

Above, $Uninsured_{is}$ is a binary indicator that respondent *i* living in state *s* was uninsured; $marst_{i,}$ is an indicator of marital status; $disability_i$ denotes disability status wasassessed through whether or not survey respondents had any type of physical and/or cognitive disability preventing work; $employed_i$ denotes current employment status; sex_i denotes sex; fpl_i is income measured in percentage points of the federal poverty level (modeled as a continuous variable); and $\Sigma_s\beta_{6,s}{*}I(state_{is}=s)$ is a vector of state fixed effects. We estimated separate models for non-Hispanic Black and Hispanic adults. We used the margins command in STATA to construct regression-adjusted estimates of uninsured rates by state, separately for non-Hispanic Black and Hispanic adults, which are displayed in the Figure of the main manuscript. Estimates were adjusted for survey weights provided with the ACS; 95% confidence intervals were estimated using standard errors clustered by state.

Second, we compared uninsured rates nationally by race, ethnicity, and state Medicaid expansion status. In national analyses (pooled across states), we compared uninsured rates among non-Hispanic Black, Hispanic, and Black adults ages 60-64 of all incomes and adults

© 2022 Patterson A et al. *JAMA Network Open.*

with incomes <138% of the FPL (eligible for Medicaid in expansion states). We estimated linear regression models of the form:

$$Uninsured_{is}=\beta_0+\beta_1 Black_{is}+\beta_2 Hispanic_{is}+\beta_3 marst_{is}+\beta_4 disability_{is}+\beta_5 employed_{is} +\beta_6 sex_{is}+\beta_7 fpl+\varepsilon_{is} \quad (2)$$

Where $Black_{is}$ and $Hispanic_{is}$ indicate non-Hispanic Black and Hispanic adults (non-Hispanic White was the reference group). The other covariates are as defined above. We interpreted β_1 and β_2 as the Black-White and Hispanic-White disparity in uninsurance. We estimated separate models for adults ages 60-64 of all incomes and adults with incomes <138% of the FPL.

We then examined how disparities in uninsured rates differed across Medicaid expansion and non-expansion states among adults ages 60-64 with incomes <138% of the FPL. To conduct these analyses, we estimated linear regression models of the form:

$$Uninsured_{is}=\beta_0+\beta_1 expand_s+\beta_2 Black_i+\beta_3 Hispanic_i+\beta_4 Black_i{*}expand_s+\beta_5 Hispanic_i{*}expand_s +\beta_6 marst_i+\beta_7 disability_i+\beta_8 employe_i+\beta_9 sex_i+\beta_{10} fpl_i+\varepsilon_{is} \quad (3)$$

where $expand_s$ is a binary indicator denoting a state's Medicaid expansion status as of December 31, 2019. Above, the regression coefficients β_2 and β_3 give the Black-White and Hispanic-White disparity in uninsurance in *non-expansion states*, while the linear combinations $\beta_2+\beta_4$ and $\beta_3+\beta_5$ give the Black-White and Hispanic-White disparity in uninsurance in *Medicaid expansion states.* Thus, β_4 and β_5 represent the difference in Black-White and Hispanic-White disparities in uninsurance in Medicaid expansion vs. non-expansion states. Estimates were adjusted for survey weights provided with the ACS; 95% confidence intervals were estimated using standard errors

© 2022 Patterson A et al. *JAMA Network Open.*

clustered by state. Estimates from models (2) and (3) are reported in the Table of the main manuscript.

© 2022 Patterson A et al. *JAMA Network Open.*
